# Taurine in morning spot urine for the useful assessment of dietary seafood intake in Japanese children and adolescents

**DOI:** 10.1186/1423-0127-17-S1-S43

**Published:** 2010-08-24

**Authors:** Mari Mori, Hideki Mori, Atsumi Hamada, Yukio Yamori

**Affiliations:** 1 Institute for World Health Development, Mukogawa Women’s University, Nishinomiya, 6638143, Japan

## Abstract

**Background:**

Since our previous report on WHO CARDIAC Study data demonstrated that 24-hour urinary (24U) taurine (Tau) excretion was a useful biomarker of seafood (SF) intake and inversely related to the mortality rates of stroke and coronary heart diseases in the world, we determined that SF is important in the risk reduction of lifestyle related-diseases. The amounts of dietary SF intake are so far estimated from a nutritional survey or 24U Tau excretion.

The sodium/potassium ratio of spot urine (SU) and the 24U ratio were reported to be significantly correlated with. Therefore, we presently examined the relationship of Tau excretion in the morning SU with 24U Tau for simplifying the population comparison and the follow-up of SF intake changes in the process of food education program (FEP).

**Methods:**

After informed consent was obtained, 54 children aged 6-11 years (Children) and 193 adolescents aged 13-18 years (Adolescents) participated in collecting precisely 24U and SU of the first urination on the same day and answered the questionnaire including age and height and weight measurements. The urine samples were measured for creatinine (Cre) and Tau, and the association of these between 24U and SU were analyzed.

**Results:**

The success rates of 24U sampling were very high in Children and Adolescents, 96.4% and 82.4%, respectively. From the result of the multiple regression analysis of SU Tau/Cre and weight we obtained formulas for predicting 24U Tau excretion in Children and Adolescents as follows: Children: 24U Tau = 16.3 (weight) + 314.3 (SU Tau/Cre) -175.2; and Adolescents: 24U Tau = 20.2 (weight) + 644.7 (SU Tau/Cre) - 569.4.

**Conclusions:**

The present study established the regression equation to estimate 24U Tau excretion from SU Tau/Cre and weight. These formulas are expected to contribute to the estimation of fish and SF intake and the follow-up of the change of the dietary behavior by FEP in Children and Adolescents.

## Background

The average life expectancy of Japanese, presently the top of the average of the males and females in the world markedly increased after World War II by the improvements in sanitation as well as in diets. Japanese school lunch system started initially to solve the problem of malnutrition by supplying flour and skimmed milk resulted later in the increased rate of the intake of meat, eggs, milk and dairy products, and Western style meals became commonly eaten by school children. This accelerated the loss of traditional Japanese dietary custom to eat rice, fish, soybean, vegetable and sea weeds [1,2]. The rapid Westernization of dietary custom increased health problem related to metabolic syndrome [3], such as obesity, hypertension, dyslipidemia and hyperglycaemia, not only in adults but also in children [4-7].

The Basic Law on Dietary Education, (“Shoku-Iku”) was enacted in 2005, to promote the well-balanced dietary custom by the education for children and their families. Although, many food educational programs (FEP) were introduced to schools and also to local communities [8], no good objective methods were available to check the improvement of the dietary custom in the children who participated in the programs.

In our world-wide epidemiological investigation, “WHO-coordinated CARDIAC (Cardiovascular Diseases and Alimentary Comparison) Study” [9,10], we could estimate the dietary intake of salt, vegetables and fruit, soy bean and sea food (SF) products by analysis of 24-hour urine (24U) excretions of the biomarkers of these diets such as sodium, potassium, isoflavones and taurine (Tau) [11-14].

Therefore, in the present study we tried to develop a new method for estimating food intake by checking morning spot urine (SU) samples instead of 24U samples to confirm the change of the dietary behavior by FEP, particularly the improvement of the intake of SF, important Japanese traditional food components by examining correlation of urinary Tau excretion, a biomarker of SF intake in 24U and the SU samples from the same individuals.

## Methods

### Study subjects

We conducted many FEP’s for children or adolescents since 2002 in Japan. The subjects of the present study were participants of FEP for elementary school children (Children) aged 6-11 years in Awaji-shima in 2002, and for junior and senior high school girls (Adolescents) aged 13-18 years in 2009, at Mukogawa Women’s University (MWU) Junior and Senior High School in Nishinomiya, Japan.

### Study design

**Figure 1:**
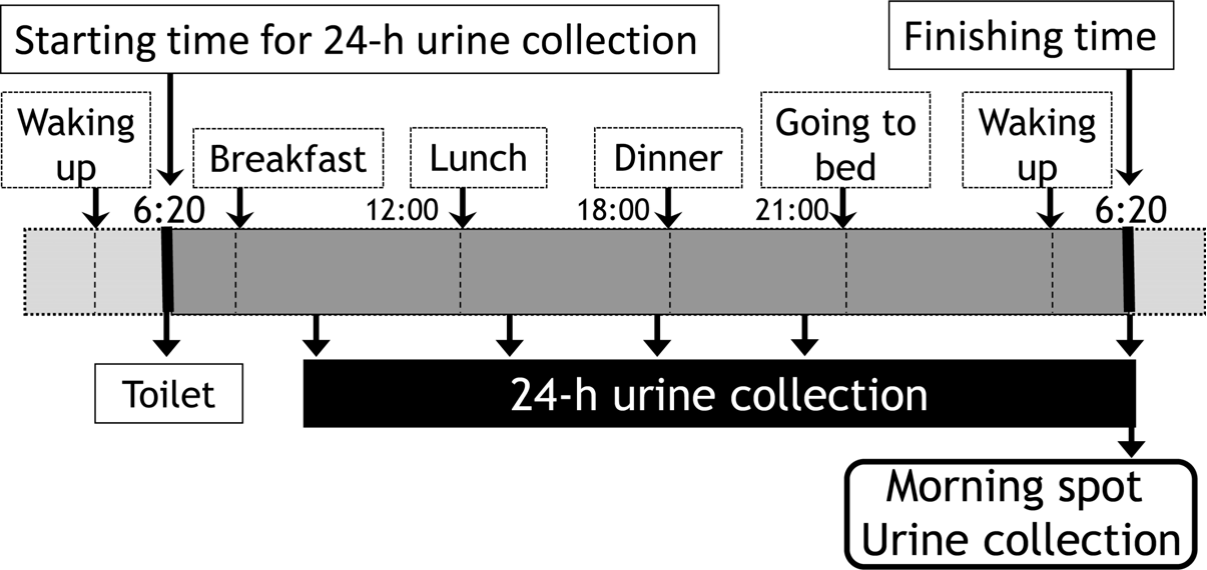
**Urine sampling method of 24-hour and morning spot urine** To void the first morning urine at toilet, for example at 6:20am, which is the starting time to collect 24U, then collecting all urine samples up until 6:20am in the next morning. SU sampling should be sampled at the last urine collection after the procedure for sampling 24U.

The protocol for collecting 24U and SU is illustrated in Fig.1. The special jar called “Aliquot cup” which we used for WHO CARDIAC Study was adopted to collect 24U by sampling one fortieth of the voided urine each time of urination [15,16]. After filling up the questionnaires including age and height and weight measurements, all participants were given aliquot-cups and instructed to collect precisely 24U by discarding the first voided urine in the morning and then collecting all urine samples up to the last urine to be sampled in the following morning at the same time when the first urine was discarded in the previous morning. The SU sample (10ml) was collected from the last morning urine sample after sampling one fortieth of the voided urine for 24U collection, and after collecting both SU and 24U samples, participants brought their samples to school. The study protocol was approved by the ethical committee of Mukogawa Women’s University, and all participants and their parents gave informed consent for the participation before enrollment.

### Urine sample analysis

After 24U volume was collected, urine samples were shared into sampling tubes for the measurement of creatinine (Cre) (mg/dl) concentration by standard laboratory methods at SRL Inc. (Tokyo, Japan) and Tau (μmol/l) were estimated by high performance liquid chromatography (HPLC) (GL Sciences, Tokyo, Japan). In order to estimate the whole 24h urinary Tau (μmol/day) excretion, we multiplied Tau concentration by the urinary volume of 24 hour. To estimate of 24U Tau from Tau/Cre ratio calculated from SU, we measured Cre and Tau concentrations in SU, and developed a formula to estimate 24U Tau excretion from checking Tau/Cre ratio in SU in Japanese Children and Adolescents.

### Statistical analysis

Statistical analysis used Stat view ver. 5.0 for Macintosh. The SU Tau/Cre ratio was determined from their respective Tau and Cre concentrations of SU. Liner forward stepwise regression analysis was employed to calculate regression equation to predict 24U Tau.  The correlation between predicted 24U Tau excretion from SU Tau excretions and actual 24U Tau excretion was then assessed. The comparison between estimated and measured values by 24U collection and sex difference in children data were carried out by student's t-test. Data were expressed as the mean ± SD, if not specified. Values of p<0.05 were considered statistically significant.

## Results

### Characteristics of the participants in this study

Characteristics of the participants are summarized in Table 1. From among 432 participants of our FEP, 56 Children (25 boys and 27 girls and 364 Adolescents (all females) tried to collect 24U, and Children and Adolescents 54 and 300, respectively could collect 24U successfully. These were very high success rates of 24U sampling in Children and Adolescents, 96.4% and 82.4% respectively. Further analysis was done in 54 Children and 193 Adolescents, who could collect both 24U and SU successfully on the same day before their participation in our FEP.

**Table 1:** Characteristics of this study participants

	Children			Adolescents
	Boys (n=25)	Girls (n=29)	Total (n=54)	(n=193)
				
Age (year)	8.2 ± 1.4	8.0 ± 1.4	8.1 ± 1.4	16.7 ± 1.3 **
Weight (kg)	31.2 ± 7.2	29.3 ± 8.6	30.2 ± 7.9	49.5 ± 6.9 **
Height (cm)	134.0 ± 11.0	131.1 ± 11.1	132.4 ± 10.1	156.2 ± 5.5 **
				
24U volume (ml)	769.3 ± 271.1	738.2 ± 289.1	752.6 ± 278.7	906.1 ± 582.4
24U Tau (μmol/day)	733.4 ± 790.9	759.4 ± 905.5	747.3 ± 847.1	1106.4 ± 630.6 **
SU Tau (μmol/l)	835.3 ± 433.2	1370.1 ± 2472.0	1122.5 ± 1840.0	1494.6 ± 1085.7
SU Cre (mg/dl)	97.0 ± 33.1	90.5 ± 40.0	93.5 ± 36.8	139.2 ± 73.9 **
SU Tau/Cre	0.99 ± 0.80	1.70 ± 0.30	1.12 ± 1.23	1.04 ± 0.69

24U: 24-h urine, SU: morning spot urine, Tau: taurine, Cre: creatinine. **:p<0.0001 vs. total of Children.

There were no significant differences between boys and girl in Children, and significant differences were noted between Children and Adolescents in age, weight, height, 24U Tau and 24U Cre (Table 1). Because of these differences, we developed different formulas for Children and Adolescents to estimate 24U Tau excretion from SU samples.

### Relationship in 24U Tau and SU Tau excretion

As shown in Table 2, among correlation coefficients of 24U Tau excretion with personal characteristics and SU variables, the highest positive correlation scores with 24U Tau excretion were variables of SU Tau/Cre in Children (R^2^=0.86) and in Adolescents (R^2^=0.69).

**Table 2:** The correlation coefficients of 24U Tau excretion with variables of personal characteristics and SU

Variable	Children (n=54)			Adolescents (n=193)	
			
	Coeff.	p value		Coeff.	p value
Age (years)	0.432	0.0013		0.103	0.1559
Weight (kg)	0.243	0.0764		0.153	0.0337
Height (cm)	0.396	0.0028		0.125	0.0835
SU Cre (mg/dl)	-0.232	0.919		-0.010	0.8956
SU Tau (μmol/l)	0.775	<0.0001		0.632	<0.0001
SU Tau/Cre	0.860	<0.0001		0.686	<0.0001

Coeff: correlation coefficients, 24U: 24-h urine, SU: morning spot urine,   Tau: taurine, Cre: creatinine.

Table 3 shows the result of the multiple liner forward stepwise regression analysis. “SU Tau/Cre and weight (R^2^=0.76)” in Children was lower determination coefficient than “SU Tau/Cre and age (R^2^=0.79)” and “SU Tau/Cre and height (R^2^=0.78)” of 24U Tau. But R^2^ is equally high, so that weight was commonly selected in both Children and Adolescents. Then we established the regression equation to predict 24U Tau excretion from SU Tau/Cre and weight in Children and in Adolescents. The obtained formulas for predicting 24U Tau excretion in Children and Adolescents were as follows;

Children: 24U Tau = 16.3(weight) + 314.3 (SU Tau/Cre) - 175.2,

Adolescents: 24U Tau = 20.2(weight) + 644.7 (SU Tau/Cre) - 569.4.

**Table 3:** Multiple regression analysis of SU Tau/Cre and weight to predict assessment   of 24U Tau excretion

	Children (n=54)								Adolescents (n=193)		
			
	***β***	p-value		***β***	p-value		***β***	p-value		***β***	p-value
SU-Tau/Cre	0.84	<0.0001		0.81	<0.0001		0.81	<0.0001		0.70	<0.0001
Weight (kg)	0.15	0.03								0.22	<0.0001
Age (year)				0.24	0.001						
Height (cm)							0.21	0.004			

R^2^	0.76	<0.0001		0.80	<0.0001		0.78	<0.0001		0.52	<0.0001
Adjusted R^2^	0.75	<0.0001		0.79	<0.0001		0.77	<0.0001		0.51	<0.0001

*β*: regression coefficient, 95%CI: the 95% confidence interval, 24U: 24-h urine, SU: morning spot urine, Tau: taurine, Cre: creatinine.

## Discussion

In the results of this study, we indicated the validity of a simple method to estimate the 24U Tau excretion by using SU Tau/Cre and weight data. There are several methods for evaluating salt intake, such as dietary recall or records from 24 to 96 hours, food frequency questionnaires or 24U collection. Generally, 24U collection is considered to be the most reliable method to evaluate salt intake [17,18]. However, in this method participants must carry urine jars for 24 hours. And sometimes participants cannot obtain urine samples precisely because of forgetting the urine collection. There have been reported some studies that evaluated the effectiveness of SU as a simple method instead of 24U collection [19-21].

In our previous “WHO CARDIAC Study”, 24U collection by using “Aliquot cups” was proven to be the useful and practical method to evaluate the different dietary habits of 61 populations in 25 countries [11-14]. In this study we reported 24U Tau excretion was positively correlated with SF intake and inversely correlated with the age adjustment mortality of coronary heart disease (CHD) and stroke [22-26]. In addition, fish and n-3 polyunsaturated fatty acid intake were reported to reduce the risk of CHD and sudden cardiac death in Western countries [27-31].

The risk of CHD was 40% lower among Japanese persons at the highest quintile of fish intake 8 times per week (180g/day) than those who ate fish once per week (23g/day) [32]. Moreover, fish intake beneficially affects the development of type 2 diabetes [33,34]. Higher total fish intake was proven to be associated with 25% reduction of diabetes [35].

On the other hand, dietary habits of Japanese children and adolescents have been Westernized dramatically in the past few decades [4], and the Westernized diet and excessive or imbalanced intake of saturated fatty acids may be important in the pathogenesis of common lifestyle-related diseases [36] and the prevalence of the risks related to metabolic syndrome increases even in preteen Japanese children [37,38].

Eating fish and SF is a recommendable dietary habit for children’s health, and morning SU collection to analyze Tau is an easy method to estimate the intake of fish and SF in children. In addition, Tau itself has been reported to be needed for the development of babies, because Tau is contained a lot in mother's milk [39], and there are many reports, that Tau is effective nutrient for the prevention of CVD [25,40], and diabetes [41]. It is important to execute FEP to make children understand good eating habits of eating fish and SF regularly which are Japanese traditional food. We think that checking SU Tau of participants in our FEP is effective for the confirmation of dietary habit improvement and for the promotion of fish and SF intake.

The limitations of the present study are the limited number of participants in Children and that only girls are studied in Adolescence. In order to apply our formulas for 24U Tau estimation widely to Children and Adolescents, we should collect 24U and SU samples from more children and also from male adolescents.

## Conclusions

The present study analyzed the association of SU Tau/Cre with 24U Tau to propose formulas to estimate 24U Tau from SU in Children and Adolescents. These formulas if utilized generally are expected to contribute to the estimation of fish and SF intake and the follow-up of the change of the dietary behavior by FEP and further to the confirmation of the health effect of fish and SF intake.

## List of abbreviations used

CARDIAC: Cardiovascular Diseases and Alimentary Comparison; 24U: 24 hour urine; Tau: taurine; SU: spot urine; Cre: creatinine; SF: seafood; HPLC: high performance liquid chromatography; FEP: food educational programs; Children: elementary school children; Adolescents: junior and senior high school girls; MWU: Mukogawa Women’s University

## Competing interests

The authors declare that they have no competing interests.

## Authors' contributions

MM designed research planning and participated in the food educational programs and analyzed the data for writing the manuscript. AH performed urinary analysis by HPLC, HM coordinated research and collected urine samples, YY was a responsible doctor for supervising human study. All authors read and approved the final manuscript. MM gave final approval of the version to be published.
